# Supplementary material for: Epistasis studies reveal redundancy among calcium-dependent protein kinases in motility and invasion of malaria parasites
Source: Nat Commun. 2018 Oct 12;9:4248. doi: 10.1038/s41467-018-06733-w (PMC6185908; doi:10.1038/s41467-018-06733-w)
Supplement: Supplementary file 1 — Supplementary Information [file 41467_2018_6733_MOESM1_ESM.pdf]

Epistasis studies reveal redundancy among calcium-dependent protein kinases in motility and invasion of malaria parasites

Fang *et al.*

Supplementary Note 1

The functional interaction between PKG and CDPK4 is conserved in *P. berghei* gametogenesis. In *P. berghei* sexual stages, PKG and CDPK4 are known to have distinct essential functions in a pathway leading to the release of male gametes in the mosquito midgut, a process called exflagellation. PKG is required for the mobilisation of intracellular calcium in response to xanthurenic acid[1], a molecule whose levels are high in insects and which is used by the parasite as a signal that it has been ingested by the mosquito[2]. Within seconds, CDPK4 acts as a calcium effector that mediates progression into S-phase of the male gametocyte and assembly of the first mitotic spindle[3-5]. As expected, the *cdpk4*-KO/*pkg*^{T619Q}-3xHA clone did not exflagellate (Supplementary Fig. 4A). Complementing this clone with *cdpk4* in its endogenous locus restored exflagellation, although using a 3xHA epitope-tagged allele of *cdpk4* did not. This was surprising since 3xHA tagging CDPK4 in a wild type background reduced exflagellation only slightly (Supplementary Fig. 4A). Tagging of CDPK4 with the same vector thus had a more severe impact on exflagellation in the *pkg*^{T619Q}-3xHA genetic background, when compared to the wild type background. We concluded that exflagellation recapitulates the gene interaction we observed in the blood stages, but since CDPK4 is essential for exflagellation, the interaction is revealed by the hypomorphic allele generated by the presence of the 3xHA tag. The exact cause of the fitness cost associated with 3xHA tagging of PKG and CDPK4 is unclear and may include alteration of protein levels, protein localisations, or kinase activities.

C2 targets both PKG and CDPK4 during *P. berghei* gametogenesis. During exflagellation, one role of PKG is to mobilise calcium, which is then thought to activate CDPK4[1]. We used this signalling paradigm to ask whether C2 targets both kinases, as the inhibitor data from *P. falciparum* had suggested. Although the T619Q mutation in PKG rendered mobilisation of calcium resistant to inhibition by C2[1], subsequent exflagellation, which requires CDPK4, remained completely sensitive (Fig. 4B). Since exflagellation in this line was not blocked by 5 µM of the unrelated PKG inhibitor Compound A (Fig. 4C), C2 must have a second target. To test whether C2 targets CDPK4 during exflagellation, we tested the

effect of C2 on a line expressing a $CDPK4^{S147M}$ allele that was previously shown to provide resistance to bumped kinase inhibitors targeting CDPK4[4]. When added prior to gametogenesis activation, C2 completely blocked exflagellation of the WT and the $CDPK4^{S147M}$ lines by inhibiting the initial PKG-dependent calcium mobilisation[1]. However, C2 no longer blocked exflagellation of the $CDPK4^{S147M}$ line when added 60 seconds after activation, when PKG is no longer required for calcium mobilisation, as opposed to the 2.34 control (Supplementary Fig. 4E-F). These data confirm that in *P. berghei*, C2 targets both PKG and CDPK4 during male gametogenesis and lends support to our interpretation of the pharmacogenetic data suggesting the *pkg-cdpk4* interactions extends to *P. falciparum* asexual blood stages.

A line expressing the $PbPKG^{T619Q}$-3xHA allele shows reduced calcium mobilisation upon gametocyte activation. PKG is required for the mobilisation of intracellular calcium in response to xanthurenic acid[1]. We thus compared the xanthurenic acid-induced calcium-dependent luminescence response of *P. berghei* gametocytes expressing the calcium-sensitive photoprotein GFP-aequorin in the wild type 2.34 control and in the *pkg*^{T619Q}-3xHA line. As observed for PfPKG, the calcium response was significantly reduced in the line expressing PKG^{T619Q}-3xHA as compared to its wild type control (Supplementary Fig. 4G) indicating that the PKG^{T619Q}-3xHA allele signals less effectively than its wild type counterpart does.

Supplementary Methods

Gametocyte production. For gametocyte production, parasites were maintained in mice phenyl hydrazine-treated three days before infection. One day after infection, sulfadiazine (20 mg/L) was added in the drinking water to eliminate asexually replicating parasites. Microgametocyte exflagellation was quantified three or four days after infection of mice by adding 4 µl of blood from a superficial tail vein to 70 µl exflagellation medium (RPMI 1640 containing 25 mM HEPES, 4 mM sodium bicarbonate, 5% FBS, 100 µM xanthurenic acid, pH 7.8). To calculate the number of exflagellation

centres per 100 microgametocytes, the percentage of RBCs infected with microgametocytes was assessed on Giemsa-stained smears. For gametocyte purification, parasites were harvested in suspended animation (SA - RPMI1640 medium containing 25 mM HEPES, 5% FCS, 4 mM sodium bicarbonate, pH 7.20) and separated from uninfected erythrocytes on a Histodenz cushion made up from 48% of a Histodenz stock (27.6% w/v Histodenz -Sigma- in 5.0 mM Tris-HCl [pH 7.20], 3.0 mM KCl, 0.3 mM EDTA) and 52% SA with a final pH of 7.2.

Calcium measurements in *P. berghei* gametocytes. WT and PKG^{T619Q}-3xHA gametocytes expressing the calcium-dependent photoprotein aequorin[1,3] were separated from uninfected erythrocytes on a Histodenz cushion. Purified gametocytes were washed 3 times in coelenterazine loading buffer (CLB - PBS, 20 mM HEPES, 20 mM Glucose, 4 mM sodium bicarbonate, 1 mM EGTA, 0.1% w/v bovine serum albumin, pH 7.2). Reconstitution was then achieved by shaking ~10^8 gametocytes, in 0.5 ml CLB, supplemented with 5 µM coelenterazine for 30 min at 19°C. Loaded gametocytes were washed twice in CLB and were then suspended in 10 ml RPMI 1640, 5% FBS, 4 mM sodium bicarbonate, pH 7.2. Prior to activation, the number of gametocytes was determined and for each replicate 100 µl containing the same number of WT or PKG^{T619Q}-3xHA gametocytes in suspended animation were injected into the same volume of ookinete medium (RPMI1640, 5 mM $NaHCO_3$, 30 µM xanthurenic acid, pH 7.4) in a 96-well assay plate of an Orion II microplate system luminometer. For each sample 50 luminescence readings were acquired over 35 seconds following activation. To compare WT and PKG^{T619Q}-3xHA calcium responses, the ratio of the areas under the curve was determined.

Supplementary Figures

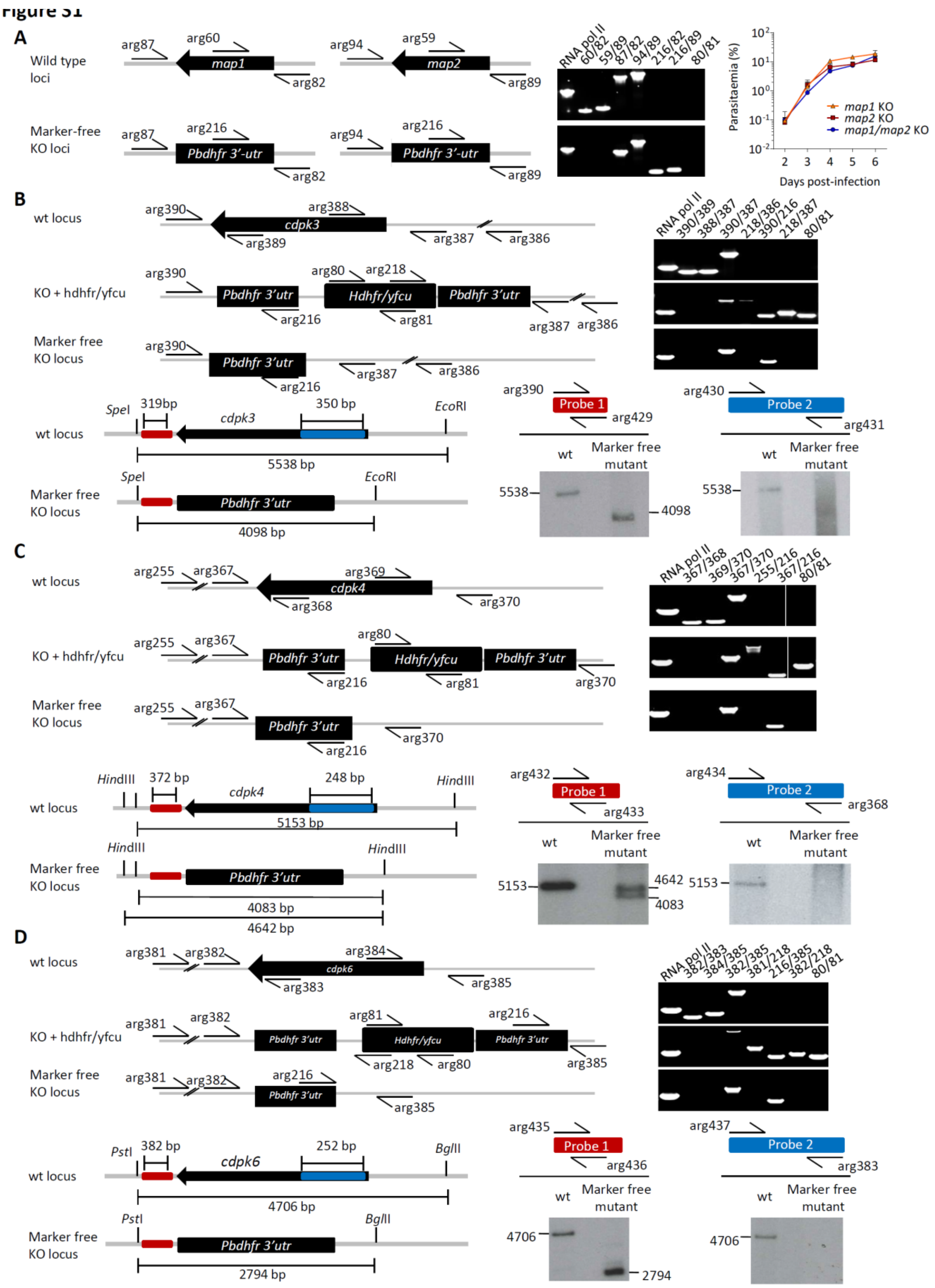

Supplementary Fig. 1. Generation of selection marker-free backgrounds to screen for genetic interactions in *P. berghei.* (A-D) Genotyping data for MAP1K-KO/MAP2K-KO, CDPK3-KO, CDPK4-KO and CDPK6-KO marker-free lines used in this study. The growth rate of MAP1K-KO/MAP2K-KO and

single KOs asexual blood stages are indicated. For CDPK3-KO, CDPK4-KO and CDPK6-KO Southern blots are shown to confirm gene deletion.

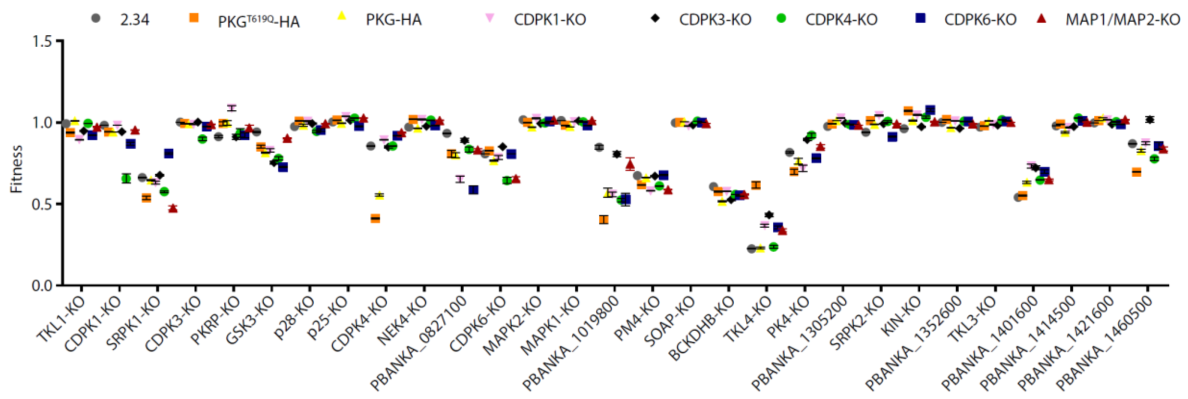

Supplementary Fig. 2. Fitness score deduced from signature-tagged mutagenesis. Fitness scores for 197 double and triple mutants showing most genes do not interact. Error bars show standard deviation from the mean from growth rate measurements in three mice.

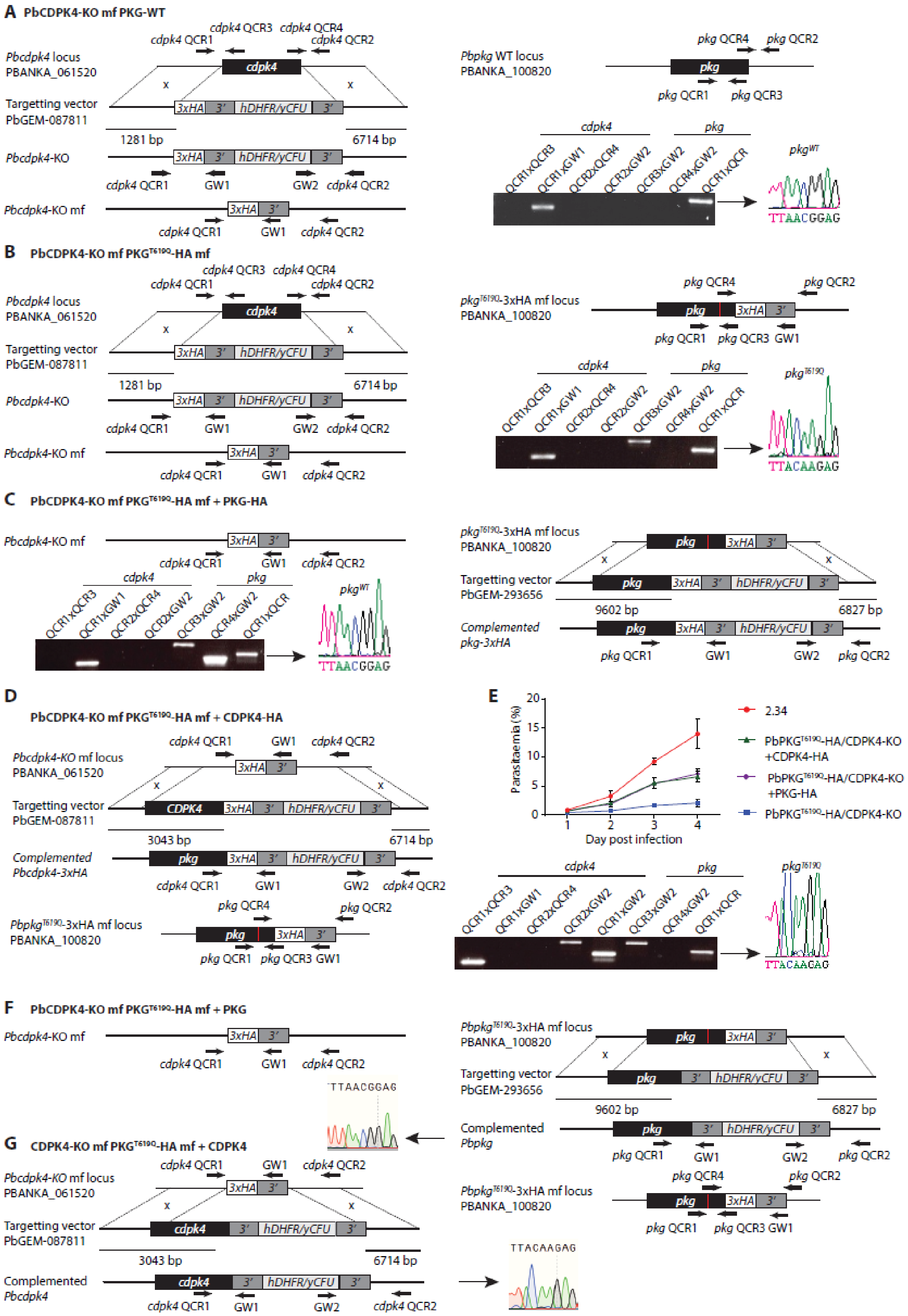

Supplementary Fig. 3. Generation and genotyping of *P. berghei* PKG and CDPK4 transgenic lines. (A-

D) Genetic modification vectors and genotyping data for *cdpk4* and *pkg* mutagenesis in *P. berghei*. Oligonucleotides used for PCR genotyping are indicated and agarose gels for corresponding PCR products from genotyping reactions are shown. For substitutions, chromatogram highlighting the mutations are also shown. (**E**) Effect of the complementation of *cdpk4* deletion or *pkg* mutagenesis with 3xHA tagged wild type alleles of *cdpk4* or *pkg* (error bars show standard deviations from 3 independent infections). (**F and G**) Genetic modification vectors and genotyping data for *cdpk4* and *pkg* complementation with non-tagged alleles of *cdpk4* and *pkg* in *P. berghei*. Oligonucleotides used for PCR genotyping are indicated and agarose gels for corresponding PCR products from genotyping reactions are shown. For substitutions, chromatogram highlighting the mutations are also shown.

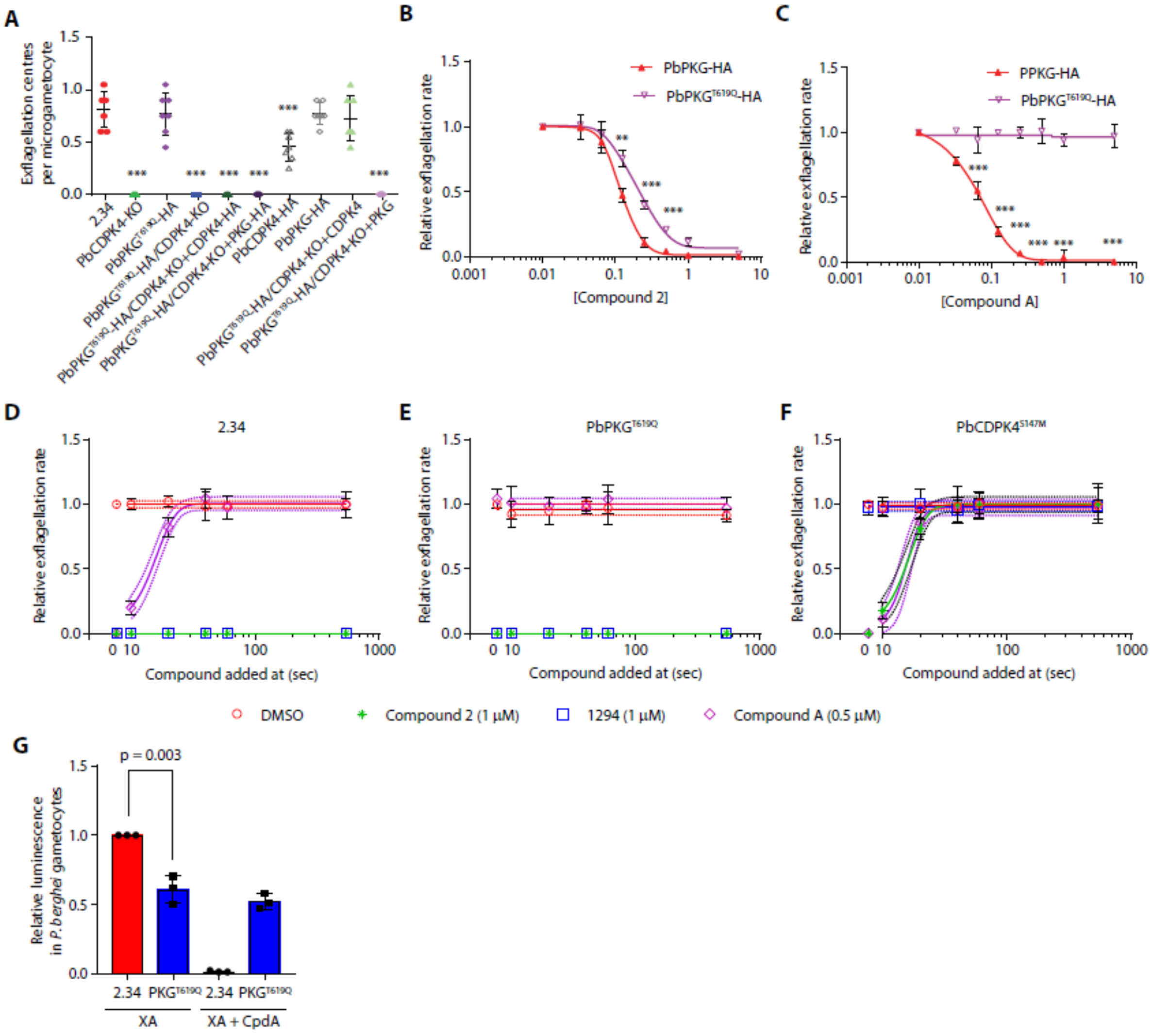

Supplementary Fig. 4. The functional interaction between CDPK4 and PKG is conserved to control *P. berghei* gametogenesis. (**A**) Effect of *cdpk4* gene deletion and/or *pkg* mutagenesis as well as complementation with 3xHA- or non- tagged wild type alleles on the exflagellation of microgametocytes (error bars show standard deviations from the mean; 6 biological replicates; one-way ANOVA, ***<0.0001). (**B**) Effect of C2 on the exflagellation of the control line PKG-3xHA and a line expressing a C2-resistant PKG^{T619Q}-3xHA allele (error bars show standard deviations from the mean; 3 biological replicates; two-way ANOVA, **<0.001, ***<0.0001). (**C**) Effect of Compound A on the exflagellation of the control line PKG-3xHA and the C2-resistant PKG^{T619Q}-3xHA line (error bars show standard deviations from the mean; 3 biological replicates, two-way ANOVA, ***<0.0001). (**D-F**) Effect of C2 and Compound A on the exflagellation of the wild type control (D) and lines expressing PKG^{T619Q}-

3xHA (E) or CDPK4^{S147M} (F) alleles when compounds are added 5 min prior to activation or at 10, 30, 60 or 540 seconds after activation with xanthurenic acid (error bars show standard deviations from the mean, biological triplicates). (**E**) Relative luminescence emitted in response to xanthurenic acid activation in the *P. berghei* 2.34 control and PKG^{T619Q}-3xHA/CDPK4-KO lines expressing the GFPaequorin calcium sensor in absence of treatment or in presence of 0.5 µM of the PKG inhibitor Compound A (error bars show standard deviations from the mean, 3 biological replicates, two-tailed t-test).

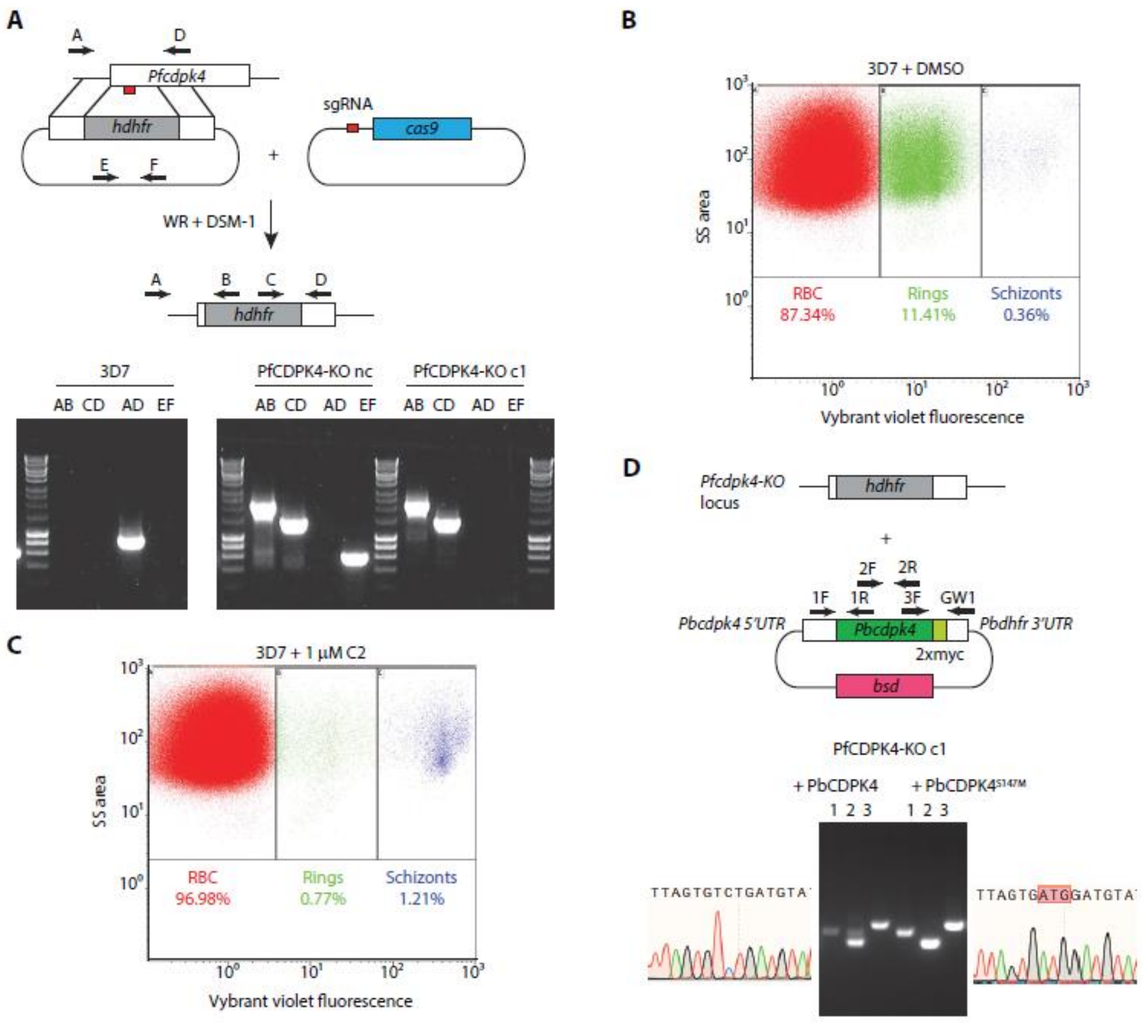

Supplementary Fig. 5. Generation, genotyping and characterisation of *P. falciparum* transgenic lines. (**A**) Genetic modification vectors and genotyping data for *cdpk4* deletion in *P. falciparum*. Oligonucleotides used for PCR genotyping are indicated and agarose gels for corresponding PCR products from genotyping reactions are shown. (**B-C**) Identification of populations of non-infected red blood cells, or Vybrant Violet stained intracellular ring and schizont parasites by flow cytometry in absence (B) or presence of C2 (C). (**D**) Genetic modification vectors and genotyping data for *cdpk4* complementation in *P. falciparum*. Oligonucleotides used for PCR genotyping are indicated and agarose gels for corresponding PCR products from genotyping reactions are shown. For the gatekeeper substitution, a chromatogram highlighting the mutation is also shown.

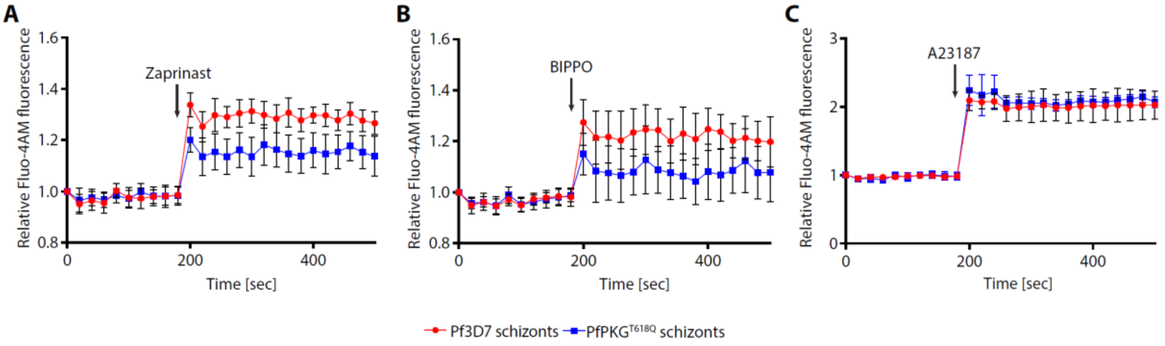

Supplementary Fig. 6. The PfPKG^{T618Q} line shows reduced Ca^{2+} mobilisation. (**A-C**) Fluorescence of Pf3D7 and PfPKG^{T618Q} synchronised schizonts loaded with the calcium indicator Fluo4-AM in response to Zaprinast, BIPPO and A23187 treatment at 180 seconds (error bars show standard deviations from the mean; 3 biological replicates, two-tailed t-test).

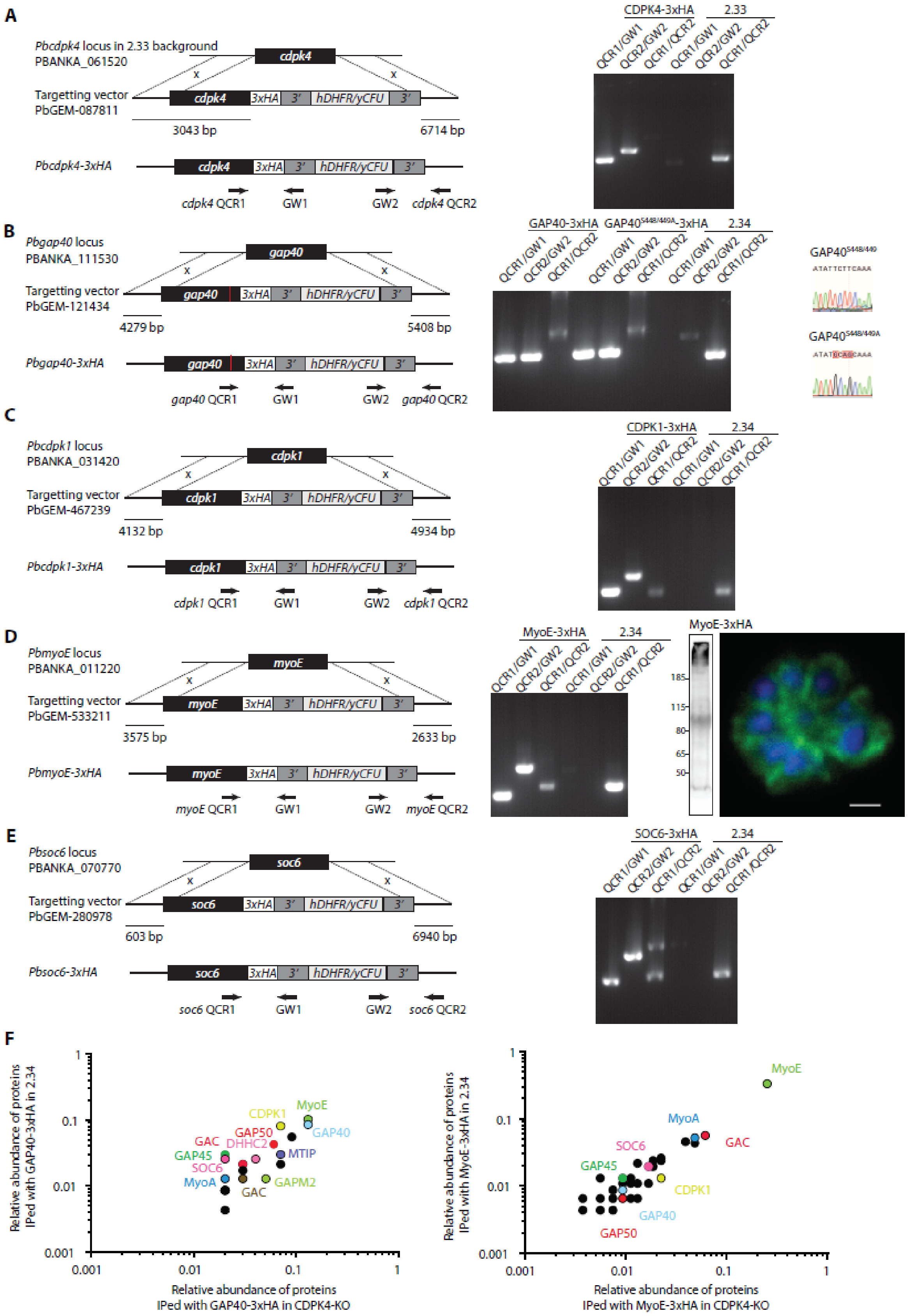

Supplementary Fig. 7. Generation, genotyping and characterisation of *P. berghei* transgenic lines aiming at investigating the function of CDPK4 substrates and interactors. (A-E) Genetic modification

vectors and genotyping data for the CDPK4-3xHA line in the 2.33 background (A), GAP40-3xHA and GAP40$^{S448/449A}$-3xHA lines (B), CDPK1-3xHA line (C), MyoE-3xHA lines (D), and SOC6-3xHA line (E). For GAP40 substitutions, chromatograms highlighting the mutations are shown. For the MyoE-3xHA western blot and immunofluorescence analyses of schizonts are shown. Scale bar is 1 µm. (**F**) Scatter plots showing the relative number of spectral counts recovered from GAP40-3xHA pull downs in the 2.34 and CDPK4-KO backgrounds (left panel) and from MyoE-3xHA pull downs in the 2.34 and CDPK4-KO backgrounds (right panel). IMC- and glideosome-associated proteins are indicated. Data is from one biological replicate.

A

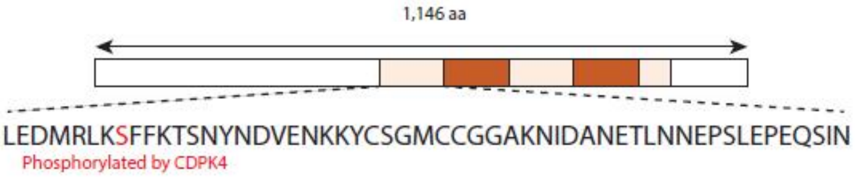
1,146 aa
LEDMRLKSFFKTSNYNDVENKKYCSGMCCGGAKNIDANETLNNEPSLEPEQSIN
Phosphorylated by CDPK4

B

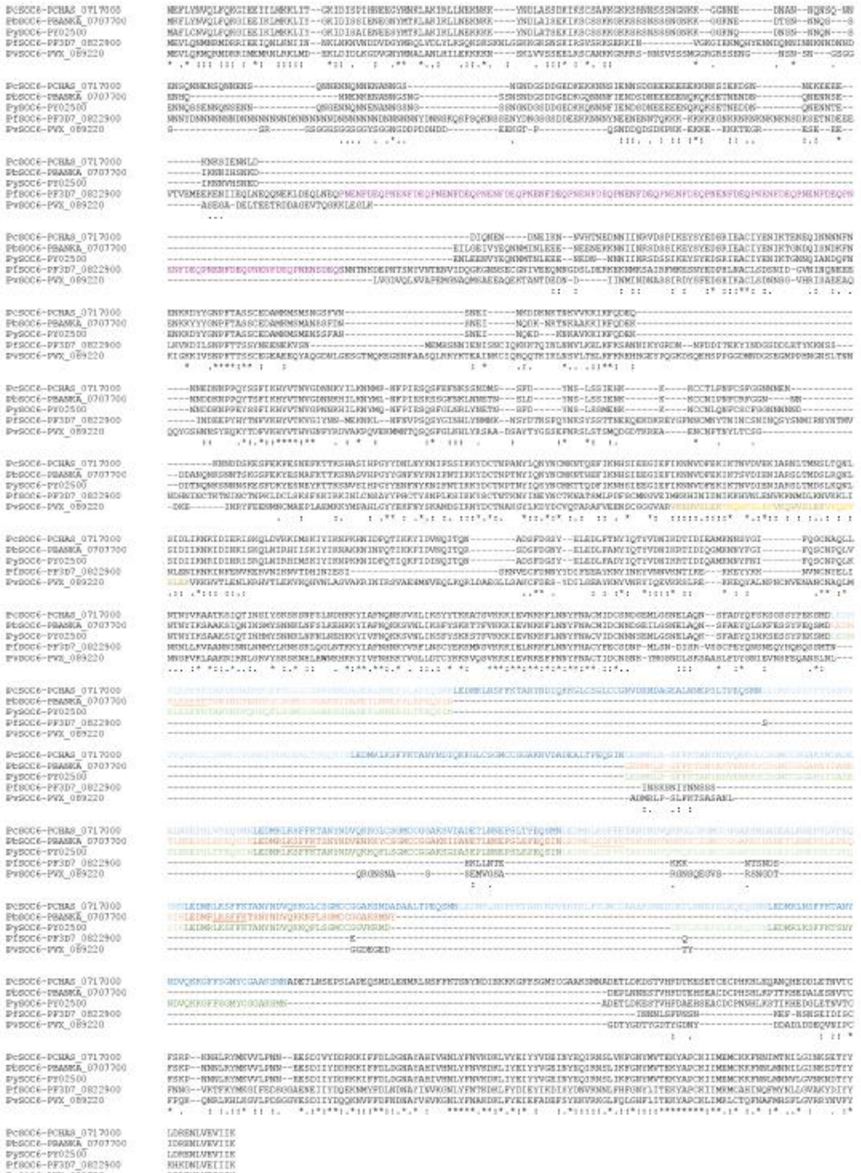

C

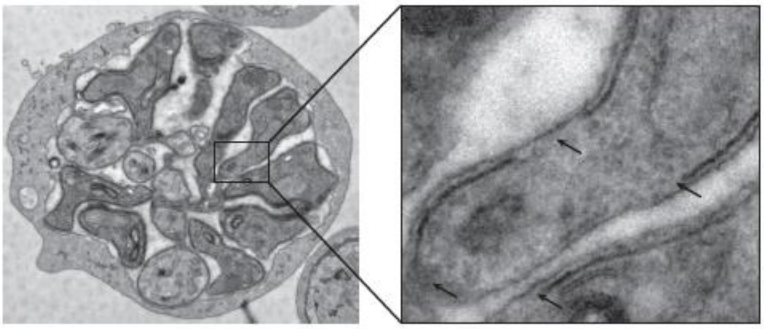

Supplementary Fig. 8. Sequence analysis of *Plasmodium* SOC6. (**A**) Overview of SOC6 structure highlighting one of the four tandem repeats. The serine residue phosphorylated by CDPK4 is highlighted in red. (**B**) Sequence alignments of SOC6 from *P. berghei*, *P. falciparum*, *P. vivax*, *P. chabaudi*, and *P. yoelii*. Tandem repeats are coloured. (**C**) Electron microscopy analysis of mature SOC6-KO schizonts, black arrows indicate gaps in the IMC that were more frequently observed in the transgenic line. Wild type control is shown in Fig. 2G.

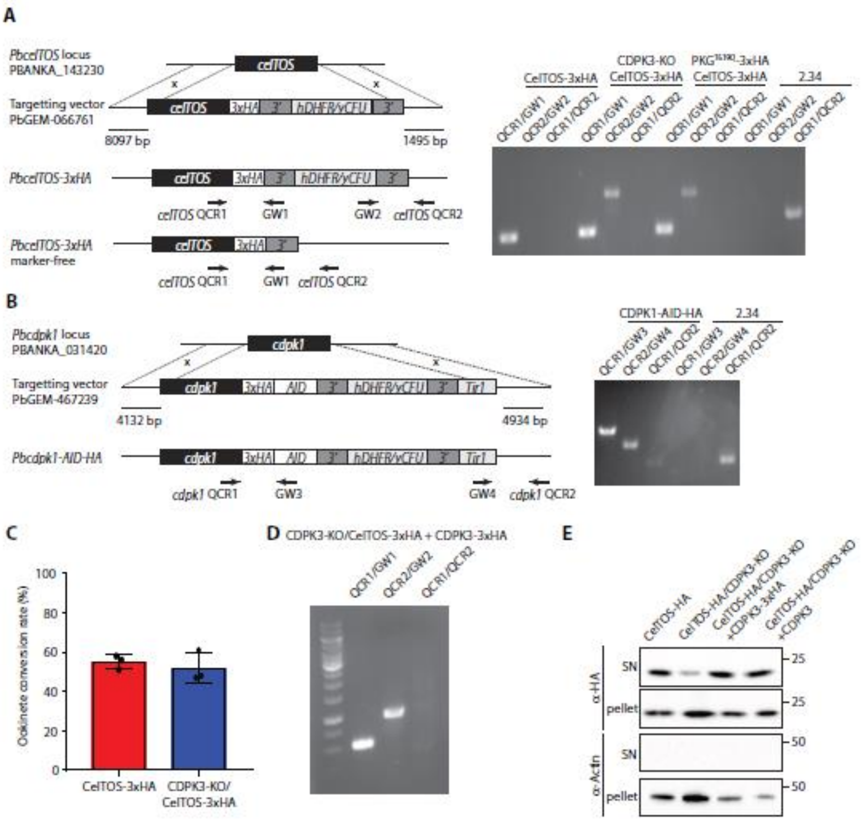

Supplementary Fig. 9. Generation of transgenic lines used for ookinete motility and secretion assays. (**A**) Genetic modification vector and genotyping data for *celtos*-3xHA tagging in *P. berghei*. Oligonucleotides used for PCR genotyping are indicated and agarose gels for corresponding PCR products from genotyping reactions are shown. (**B**) Genetic modification vector and genotyping data for *cdpk1*-AID/HA tagging in *P. berghei* and insertion of a cassette for the expression of the Tir1 protein under the control of the *hsp70* promoter and the 3'UTR of *p28*. Oligonucleotides used for PCR genotyping are indicated and agarose gels for corresponding PCR products from genotyping reactions are shown. (**C**) Ookinete conversion rate of CelTOS-3xHA and CDPK3-KO/CelTOS-3xHA lines (error bars are standard deviations from the mean, three independent ookinete cultures, two-tailed t-test). (**D**) Genetic modification vector and genotyping data for *cdpk3*-KO/*celtos*-3xHA complementation with *cdpk3*-3xHA tagging in *P. berghei* using PlasmoGEM vector PbGEM-084520. Oligonucleotides for *cdpk3* locus genotyping are indicated and agarose gels for corresponding PCR products from genotyping reactions are shown. (**E**) Complementation of the CDPK3-KO/CelTOS-3xHA line with a CDPK3-3xHA

allele or with a PlasmoGEM artificial chromosome containing *cdpk3* and its UTRs (PbAC02-20d) restores secretion of CelTOS-3xHA in ookinetes.

Figure 3A

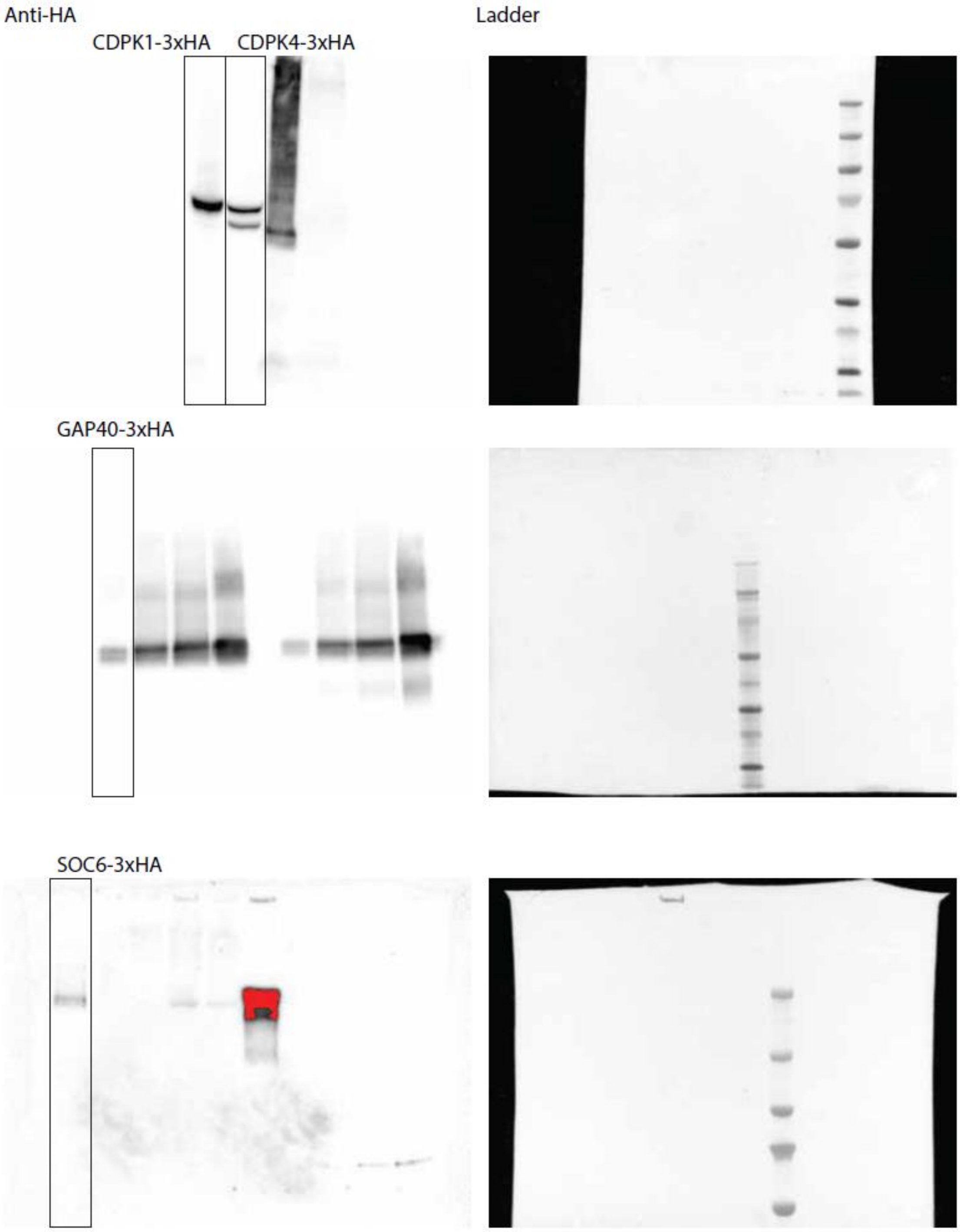

Figure 5C

Anti-HA

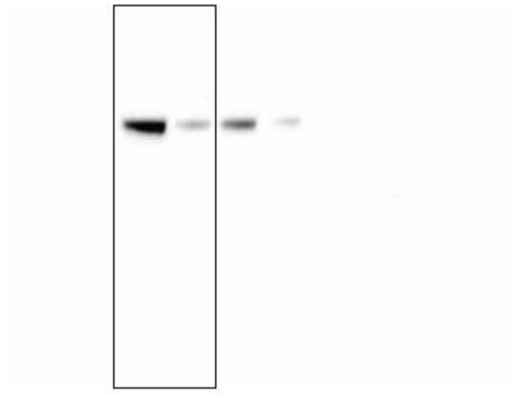

Ladder

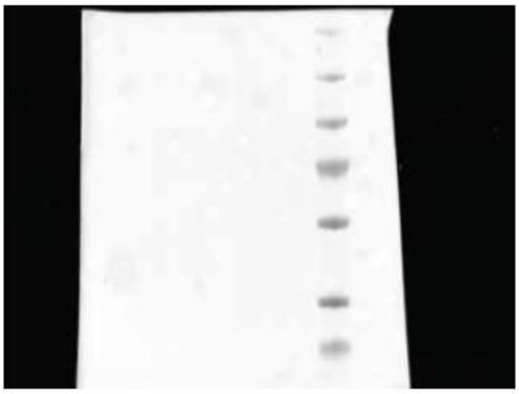

Anti-Actin

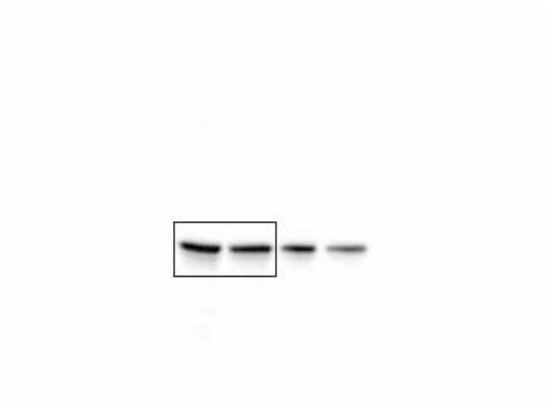

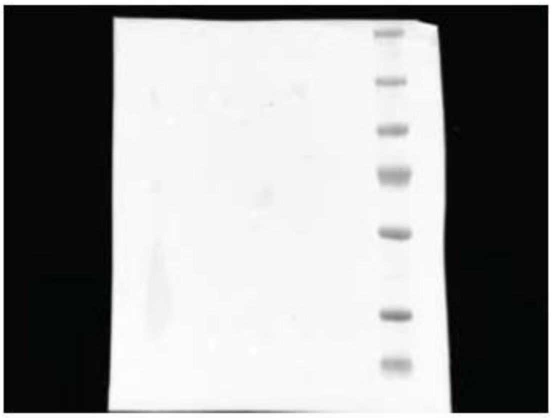

Figure 5E

Anti-HA Supernatant

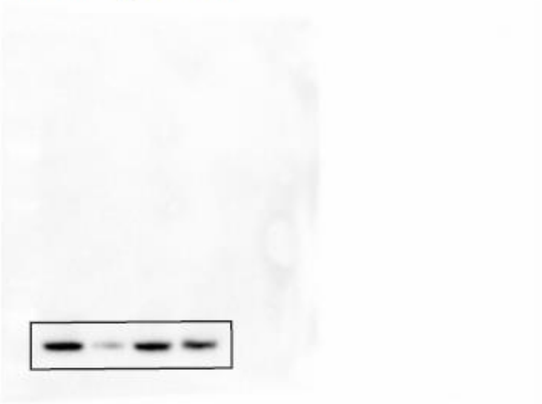

Ladder

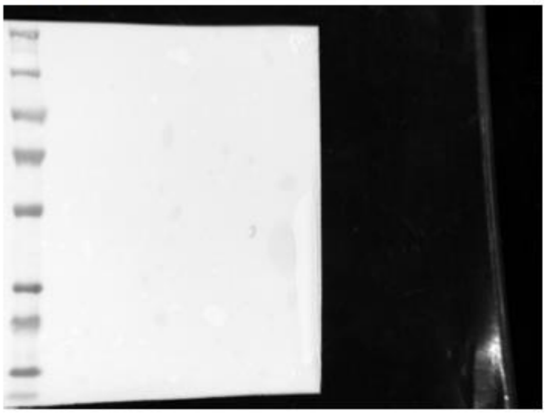

Anti-HA Pellet

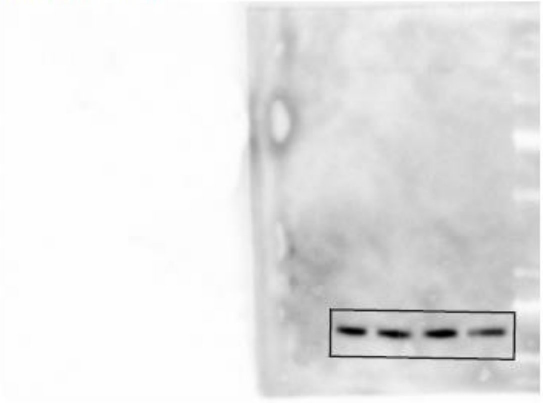

Ladder

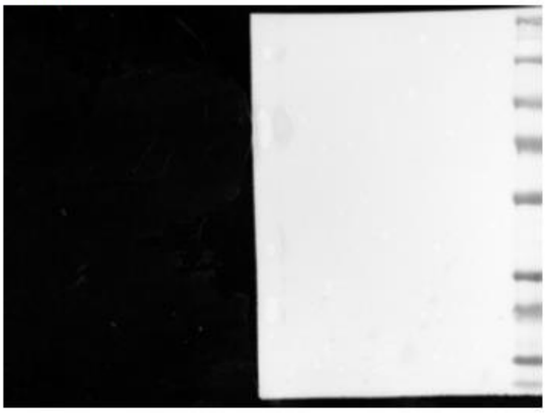

Anti-Actin

Pellet Supernatant

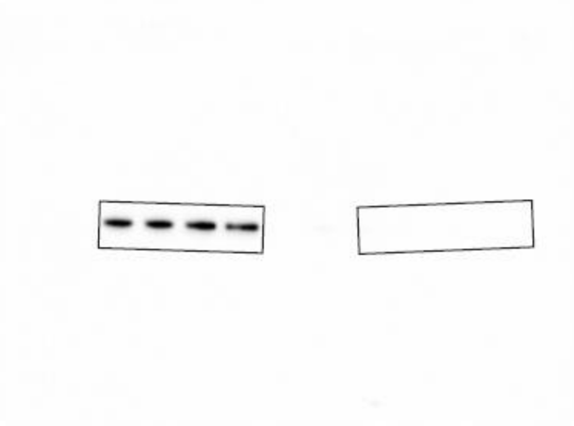

Ladder

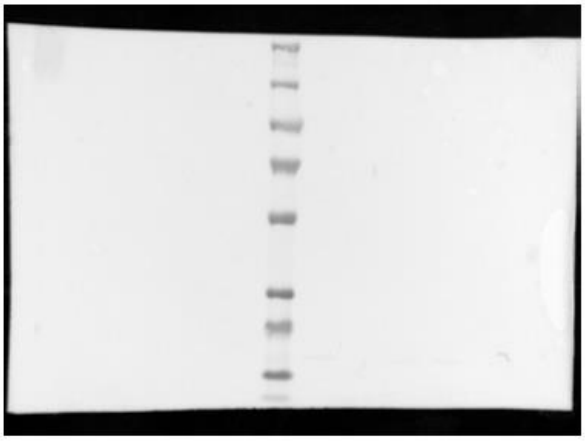

Figure 5F

Anti-HA Supernatant

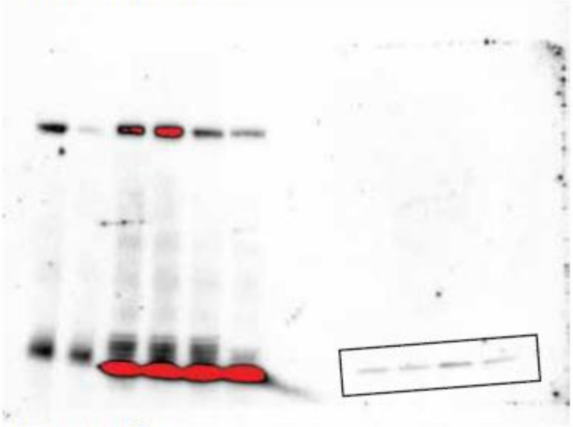

Ladder

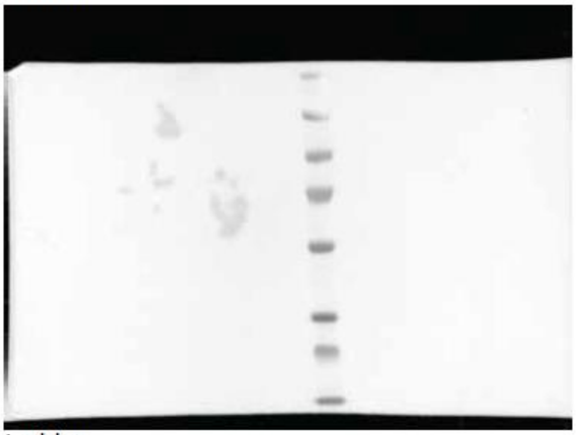

Anti-HA Pellet

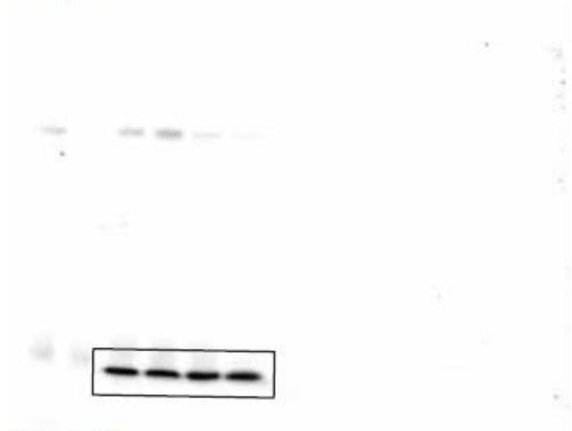

Ladder

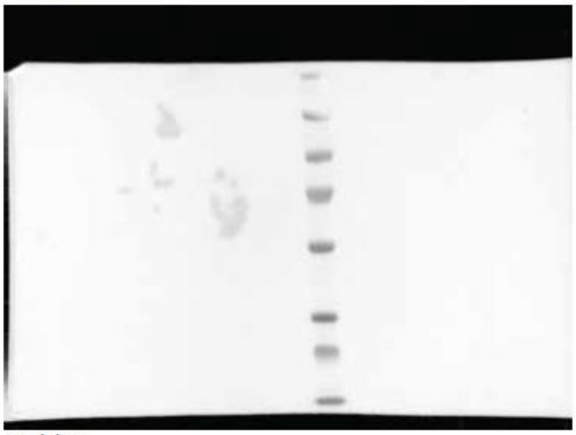

Anti-Actin

Pellet Supernatant

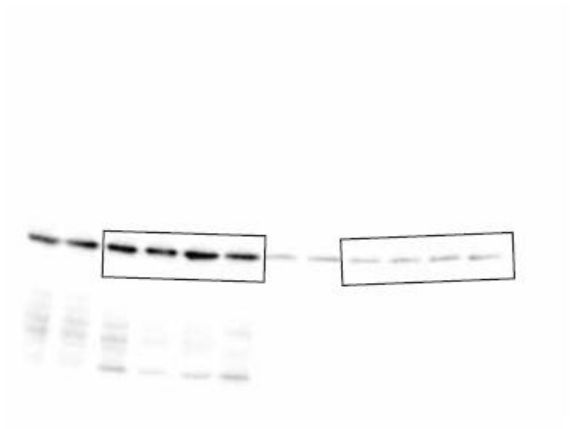

Ladder

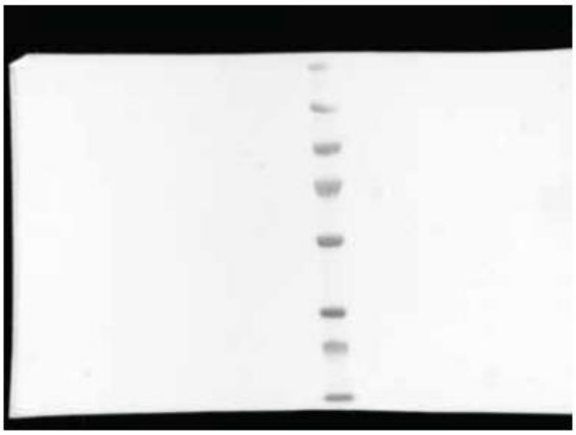

Figure 5H

Anti-HA Supernatant

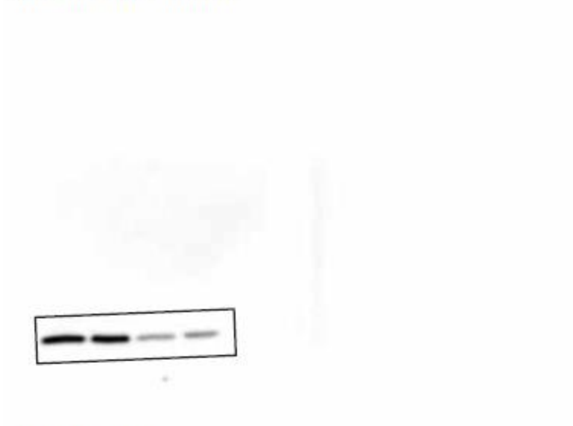

Ladder

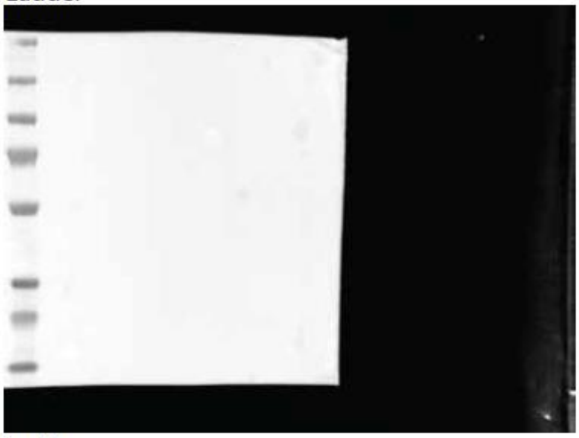

Anti-HA Pellet

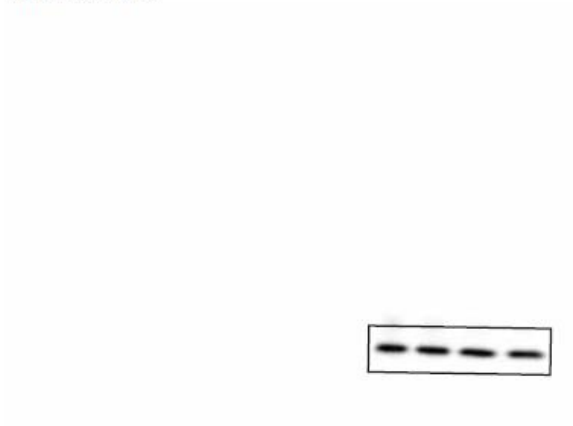

Ladder

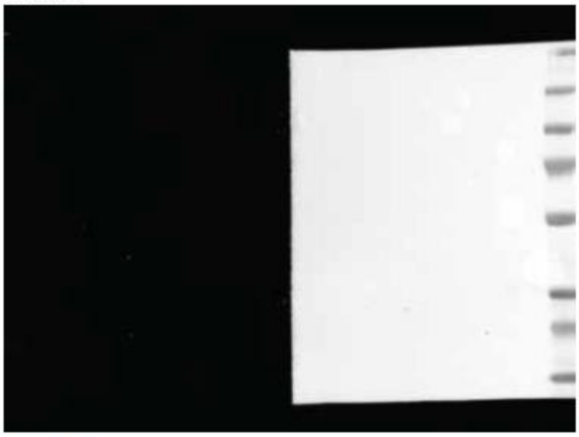

Anti-Actin

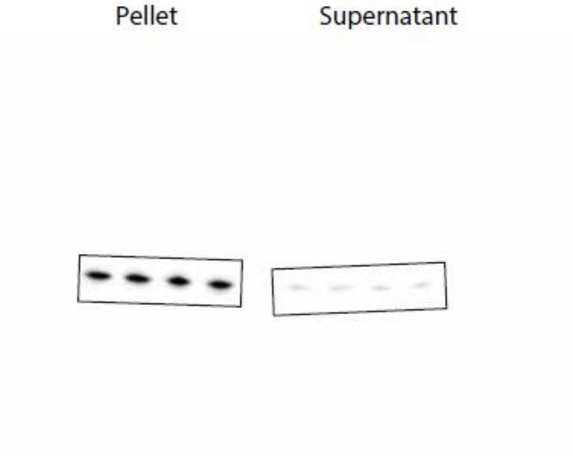

Ladder

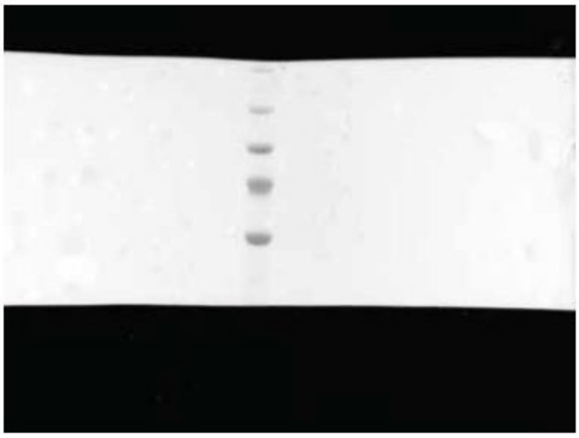

Supplementary Figure 9E

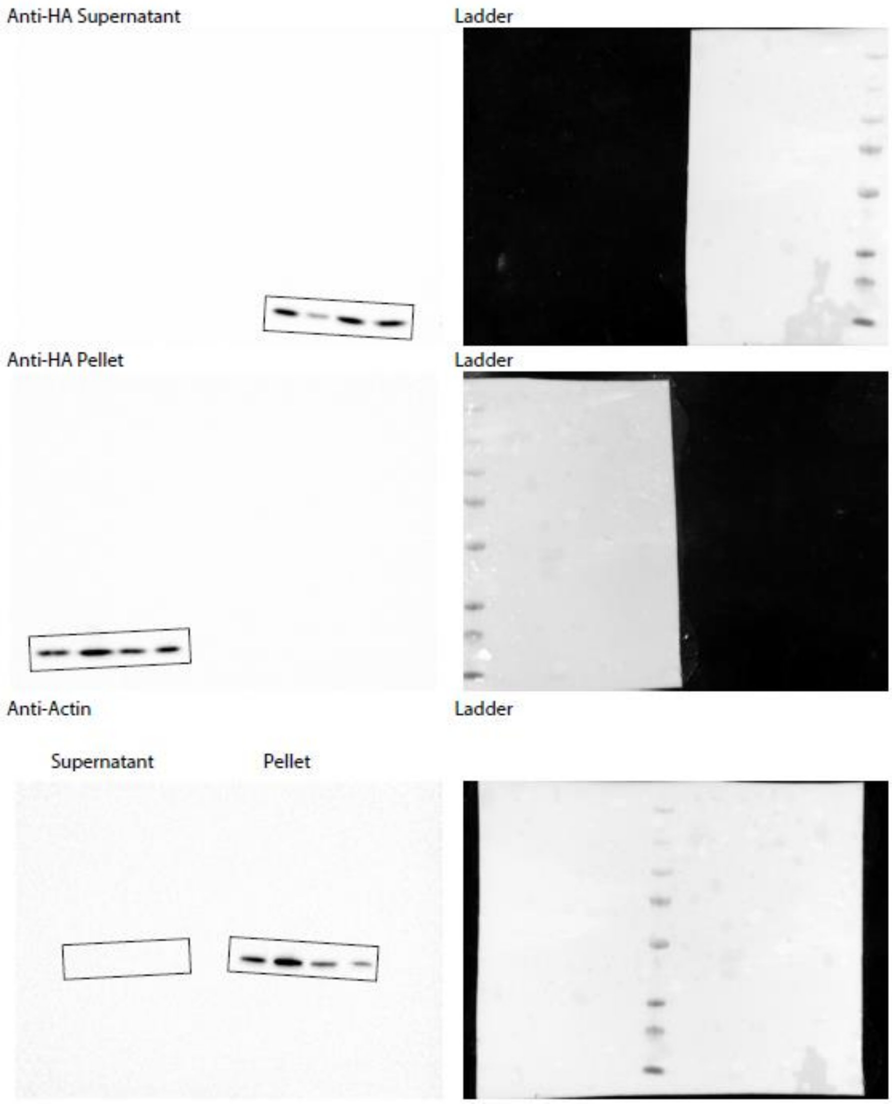

Supplementary Fig. 10. Western blot source files

Supplementary References

1 Brochet M, Collins MO, Smith TK, Thompson E, Sebastian S, Volkmann K, Schwach F, Chappell L, Gomes AR, Berriman M, Rayner JC, Baker DA, Choudhary J & Billker O. Phosphoinositide metabolism links cGMP-dependent protein kinase G to essential Ca^{2+} signals at key decision points in the life cycle of malaria parasites. *PLoS Biology* **12**, e1001806, (2014).

2 Billker O, Lindo V, Panico M, Etienne AE, Paxton T, Dell A, Rogers M, Sinden RE & Morris HR. Identification of xanthurenic acid as the putative inducer of malaria development in the mosquito. *Nature* **392**, 289-292, (1998).

3 Billker O, Dechamps S, Tewari R, Wenig G, Franke-Fayard B & Brinkmann V. Calcium and a calcium-dependent protein kinase regulate gamete formation and mosquito transmission in a malaria parasite. *Cell* **117**, 503-514., (2004).

4 Fang H, Klages N, Pardo M, Yu L, Choudhary J & Brochet M. Multiple short windows of CDPK4 activity regulate distinct cell cycle events during *Plasmodium* gametogenesis. *Elife*, e26524, (2017).

5 Invergo BM, Brochet M, Yu L, Choudhary J, Beltrao P & Billker O. Sub-minute Phosphoregulation of Cell Cycle Systems during *Plasmodium* Gamete Formation. *Cell Rep.* **21**, 2017-2029, (2017).
