# Supplementary material for: Epistasis studies reveal redundancy among calcium-dependent protein kinases in motility and invasion of malaria parasites
Source: Nat Commun. 2018 Oct 12;9:4248. doi: 10.1038/s41467-018-06733-w (PMC6185908; doi:10.1038/s41467-018-06733-w)
Supplement: Supplementary file 3 — Description of Additional Supplementary Files [file 41467_2018_6733_MOESM3_ESM.pdf]

Description of Additional Supplementary Files

File Name: Supplementary Data 1
Description: PlasmoGEM vectors used in this study.

File Name: Supplementary Data 2
Description: Interaction coefficients and associated *p*-values determined from the genetic interaction screen.

File Name: Supplementary Data 3
Description: List of proteins and number of corresponding unique peptide counts identified in immunoprecipitates.

File Name: Supplementary Data 4
Description: List of oligonucleotides used in this study.
